# Reasons for inpatients not to seek clarity at Dr George Mukhari Academic Hospital, Pretoria

**DOI:** 10.4102/phcfm.v6i1.576

**Published:** 2014-03-10

**Authors:** Langalibalele H. Mabuza, Olufemi B. Omole, Indiran Govender, John V. Ndimande

**Affiliations:** 1 Department of Family Medicine and Primary Health Care, University of Limpopo (Medunsa Campus), South Africa; 2 Department of Family Medicine, University of the Witwatersrand, South Africa

## Abstract

**Background:**

Healthcare practitioners should provide patients with information regarding their clinical conditions. Patients should also feel free to seek clarity on information provided. However, not all patients seek this clarity.

**Objectives:**

To explore the reasons inpatients gave for not seeking clarity on information that was received but not understood.

**Methods:**

This was a qualitative arm of a larger study, titled ‘Are inpatients aware of the admission reasons and management plans of their clinical conditions? A survey at a tertiary hospital in South Africa’, conducted in 2010. Of the 264 inpatients who participated in the larger study, we extracted the unstructured responses from those participants (*n* = 152) who had indicated in the questionnaire that there was information they had not understood during their encounter with healthcare practitioners, but that they had nonetheless not sought clarity. Data were analysed thematically.

**Results:**

Themes that emerged were that inpatients did not ask for clarity as they perceived healthcare practitioners to be ‘too busy’, aloof, non-communicators and sometimes uncertain about patients’ conditions. Some inpatients had unquestioning trust in healthcare practitioners, whilst others had experiences of bad treatment. Inpatients had poor self-esteem, incapacitating clinical conditions, fear of bad news and prior knowledge of their clinical conditions. Some inpatients stated that they had no reason for not seeking clarity.

**Conclusion:**

The reasons for not seeking clarity were based on patients’ experiences with the healthcare practitioners and their perceptions of the latter and of themselves. A programme should be developed in order to educate inpatients on effective communication with their healthcare practitioners.

## Abstrait

**Les raisons pour lesquelles les patients internes de l'hôpital Dr George Mukhari Academic, à Pretoria, ne demandent pas d'explications concernant leur état**

**Présentation:**

Les professionnels de la santé devraient fournir à leurs patients des informations concernant leur état clinique. Les patients devraient également se sentir libres de demander des explications quant aux informations qui leurs sont fournies. Cependant, tous les patients ne le font pas.

**Objectifs:**

Étudier les raisons fournies par les patients pour expliquer le fait qu'ils ne demandent pas d'explications concernant les informations reçues mais non comprises.

**Méthodes:**

Cette étude constitue un volet qualitatif d'une étude de plus grande envergure, intitulée « Les patients internes ont-ils conscience des raisons de leur admission et des plans de prise en charge de leur état clinique? Étude réalisée dans un hôpital tertiaire en Afrique du Sud », réalisée en 2010. Sur les 264 patients internes qui ont participé à l'intégralité de l’étude, nous avons extrait les réponses non structurées des participants (*n* = 152) qui avaient indiqué dans le questionnaire qu'ils n'avaient pas compris certaines informations fournies lors de leurs échanges avec les professionnels de la santé, et n'avaient néanmoins pas demandé d'explications. Les données ont été analysées par thème.

**Résultats:**

Les thèmes qui en sont ressortis étaient que si les patients internes n'avaient pas demandé d'explications, c'est parce qu'ils avaient le sentiment que les professionnels de la santé étaient « trop occupés », distants, communiquaient mal, et parfois n’étaient pas certains de l’état des patients. Certains patients avaient une confiance aveugle en eux, mais d'autres avaient déjà été mal traités. Les patients internes avaient une piètre estime d'eux-mêmes, souffraient d'un état clinique débilitant, avaient peur d'entendre de mauvaises nouvelles ou connaissaient déjà leur état clinique. Certains patients ont affirmé qu'ils n'avaient aucune raison de demander des explications.

**Conclusion:**

Les raisons pour lesquelles les patients internes ne demandaient pas d'explications s'appuyaient sur leurs expériences avec les professionnels de la santé et leur perception de ces derniers et de leur propre personne. Il conviendrait d’élaborer un programme visant à éduquer les patients internes afin de leur permettre de mieux communiquer avec les professionnels de la santé.

## Introduction

Countries world-wide have adopted and adapted The Patients’ Rights Charter which seeks to address comprehensive healthcare for patients.^1–4^ Although a patient's right to access information has been assured in each country's charter, the realisation of this ideal is in the hands of individual healthcare institutions.

It is the right of every patient whose clinical condition warrants admission to a healthcare institution to be made aware of the clinician's working diagnosis, the reason for the admission and the inherent risks of non-admission. Once in the ward, the patient needs to be updated constantly with regard to the management plan, including the estimated length of hospital stay, investigative procedures, medication and any operative procedures envisaged. It has been shown in patients with tuberculosis that raising awareness about the patient's condition improves patient cooperation with healthcare practitioners^5^ and guides them toward realistic expectations with regard to the healthcare team's abilities.^6^ On the other hand, patients who receive poor communication from their physicians have been found to have a 19% higher risk of non-adherence to treatment, compared with those who received adequate communication.^7^

Acquisition of information by a patient is the responsibility of both the healthcare practitioner and the patient.^5^ A balance is required between the responsibility of the healthcare practitioner to inform patients and the responsibility of the patients to seek information and clarification where the latter did not understand what was said. The healthcare practitioner can only be guided by the patient to areas that require emphasis and clarification. This has been found to occur in a balanced healthcare practitioner–patient relationship^8^ and such effective communication has been found to improve patient health outcomes.^9^ Patient engagement with healthcare practitioners about their health conditions has been found to benefit patients as they take charge of their own health.^10–12^ However, anecdotal evidence suggests that patients do not always seek clarification on health issues from their healthcare practitioners, for a variety of reasons. The fact that patients do not engage with their healthcare practitioners so as to seek clarification about their clinical conditions prompted the authors to conduct a study amongst inpatients at Dr George Mukhari Academic Hospital (DGMAH). This study sets out to explore the reasons given by inpatients for not seeking clarity on information received from their healthcare practitioners.

### Contribution to field

It has been established that effective communication with patients improves patient adherence to management.^7^ For patients to adhere, they must understand the information given and to understand, they need clarity. However, patients are often reluctant to engage with healthcare practitioners in a conversation about their clinical condition, no matter how little they understand.^13^ The reasons for this have not been explored, thus this article seeks to bridge that gap.

## Research method and design

### Design

This study was the qualitative arm of the study titled ‘Are inpatients aware of the admission reasons and management plans of their clinical conditions? A survey at a tertiary hospital in South Africa’, conducted from 6–17 December 2010 on 264 patients.

### Setting

The study was conducted at DGMAH, a tertiary hospital located about 30 km north of the city of Tshwane (Pretoria). It serves as the training hospital for health sciences students at the University of Limpopo (Medunsa Campus). It comprises 39 wards which are clustered according to clinical disciplines. The hospital is a 1550-bedded hospital, with an average daily bed occupancy rate of 65.5% (982 inpatients) at the time of the study.

### Sampling

The study population comprised all inpatients at the time of the study excluding those in the paediatric ward, psychiatric ward, labour ward, burns unit and intensive care wards. These wards were excluded because of the difficulty associated with obtaining informed consent from these patients. There were 27 remaining wards after these exclusions, with an average of 31 patients in each. Accordingly, that amounted to a study population of 837.

At a confidence level of 95% and a confidence interval of 0.05, the sample size from the study population mentioned above worked out to 264.^14^ Since the sample size represented 32% of the total study population, the number of patients from each ward was calculated *pro rata* using this percentage. The patients were selected using a systematic sampling method; for example, in a ward with 25 patients we selected eight patients (32%), where the first patient was selected by throwing a dice and every third patient thereafter was selected from the list in the ward patient register. If a patient declined participation, or was clinically or mentally unstable, they were excluded and the next patient in the patient register was given the option to participate.

Apart from collecting sociodemographic information and other structured answers, the questionnaire focused on patients’ perceptions and views on issues pertaining to awareness of the reasons for their admissions and management plan. One of these questions was:

You have stated that you have the responsibility as a patient to ask for clarity where you did not understand what you were told. You have also stated that there was something about your illness and/or condition which when you were informed you did not understand. What would you say led you not to seek this clarity?

Participants’ unstructured responses to this question were extracted. There were 152 respondents for this question.

### Data collection methods

A questionnaire was developed *de novo* by the researchers, subjected to peer review by an independent researcher and a statistician and then piloted in a nearby 158-bedded hospital. It was translated from English into Setswana and isiZulu (the predominant languages spoken at the research setting) and then back-translated into English to ensure the accuracy of the translation. Each consenting patient was given a questionnaire by one of the five research assistants to complete in their preferred language. Privacy was ensured by conducting the data collection session with curtains drawn around the patient's bed. The research assistant was present for each patient to assist with clarification of any question on the questionnaire. The responses to the semi-structured questionnaire given in the local languages were translated into English through the services of an expert in Linguistics.

### Data analysis

After initial analysis of the participants’ responses and themes developed therefrom, the findings of this study were subjected to researcher triangulation amongst the four researchers. Group consensus on themes and categories was also achieved at a group meeting. An independent senior researcher did a peer review and acted as a ‘devil's advocate’, challenging the research team to provide evidence for their interpretations and the conclusions drawn, based on the data.^15^ The research team subjected the data to an iterative process, whereby the data were visited and revisited, connecting them with emerging insights and progressively refining the focus of understanding.^16^ In this way, data gathered were analysed using thematic analysis.^17^ Member checking to authenticate the study findings through eliciting feedback from the participants^18^ was not performed because of a high patient turnover in a number of the wards in which the study was conducted.

### Trustworthiness

To ensure trustworthiness of the study findings, the principles of credibility, dependability, confirmability and transferability were followed.^19^ To ensure data credibility, the research team checked the data entry of each questionnaire to ensure its correctness. Furthermore, the study findings were subjected to peer review by a senior researcher who was not part of the study.^19^ With regard to dependability, we looked at the range of experience (variability) rather than the average experience of all the participants.^20^ Therefore, each participant's response was considered when grouping the data categories into themes. Confirmability was ensured by non-involvement of the research team in data collection, as well as by researcher triangulation so as to achieve objectivity in data collection and reporting.^19^ Transferability was ensured by providing a thick description of the study to allow evaluation regarding how well the study conclusions can be applied to other similar settings.^21^

## Results

We identified seven healthcare practitioner-related and five patient-related themes (see Table 1). These are presented below with supporting quotations where applicable.

**TABLE 1:** List of themes.

A. Healthcare practitioner-related themes	B. Patient-related themes
1. Healthcare practitioners were perceived to be too busy	1. Notion that healthcare practitioners held patients in low esteem
2. Experiences of bad treatment	2. Incapacitating clinical condition
3. Patients forbidden to ask questions	3. No reason for not asking
4. Healthcare practitioners were perceived to be aloof from patients	4. Patients’ fear of bad news
5. Unquestioning trust in health care practitioners	5. Patients already knew their conditions
6. Language barrier	-
7. Healthcare practitioners were feared by patients	-
8. The healthcare practitioner appeared uncertain about the patient's condition	-

### Healthcare practitioner-related themes

#### Theme 1: Healthcare practitioners were perceived to be too busy

The inpatients reported that they were attended to by healthcare practitioners who appeared to be very busy during the ward rounds, moving speedily from one patient to another. Information would be given through the nurse interpreter without eliciting questions from the patient to test their understanding of the information given:

‘It was hard enough to ask questions initially because he seemed really busy, so…I just chose not to ask anything again’. (Female, aged 13–20, with tertiary education)

‘Nurses don't want to explain anything to patients. Always busy’. (Female, aged 13–20, with secondary education)

#### Theme 2: Experiences of bad treatment

Healthcare practitioners were perceived to be ill-disposed toward the inpatients. This perception affected the inpatients’ willingness to engage the healthcare practitioners about their clinical conditions:

‘Here in this hospital it isn't easy, as the nurses treat us so bad, so you end up not able to ask anything’. (Male, aged 51–60, with secondary education)

‘The nurses are so impatient with us and it is sad because we need their help. What they say goes. We don't even have a chance to ask anything without being snapped at’. (Female, aged 21–30, with tertiary education)

This perception led some patients to pretend they understood all the information they received, as they saw no benefit in engaging the healthcare team any further:

‘How can you ask anything about your condition when they don't even care if you're cold and need help to bath, so I'll just pretend to be fine….You just don't receive proper help and care’. (Male, aged 41–50, with secondary education)

#### Theme 3: Patients forbidden to ask questions

Some patients reported that they were advised not to ask questions, whilst others were told that the healthcare practitioners knew better and that they were not to be bothered with questions:

‘It's as if they are pressing you or stopping you from asking questions. They tell you that they know better so you shouldn't ask any questions’. (Male, aged 31–40, with secondary education)

‘Doctor X tells us that he's a doctor and he knows best so we shouldn't bother him with questions and that's not right because it's my body and my condition. I'm the one in pain and need answers and assurance’. (Female, aged 31–40, with secondary education)

#### Theme 4: Healthcare practitioners were perceived to be aloof

The patients reported that healthcare practitioners were inaccessible because they did not communicate with patients, but rather amongst themselves:

‘They are so distant that you end up keeping quiet and sometimes when you respect someone a lot you end up having to keep quiet when you shouldn't’. (Female, aged 21–30, with tertiary education)

‘Doctors just discuss among themselves’. (Female, aged 21–30, with secondary education)

‘It's not that I'm slandering their name but they hardly ever tell us anything so it's up to them if they tell us anything’. (Male, aged > 60, with secondary education)

Patients also found it difficult to approach the healthcare practitioners, who were perceived to be distant:

‘Negative, unapproachable health workers’. (Male, aged 51–60, with secondary education)

#### Theme 5: Unquestioning trust in the healthcare workers

Some inpatients saw healthcare practitioners, specifically medical doctors, as knowing what they were doing. Some inpatients reported that they saw no reason to understand what the doctors were doing and others reported that they had a good relationship with the healthcare workers:

‘I just thought that my doctor knows best and indeed I'm happy about how I'm treated by all the doctors and nurses’. (Female, aged 51–60, with primary education)

‘Sometimes I don't understand what the doctors or nurses say but I don't ask because I trust that they know what they are doing and it is for my own good’. (Male, aged 41–50, with no formal education)

‘I wait for doctors to tell me how I am progressing, although I do not understand, at times I do not find my understanding necessary. They know what they are doing’. (Male, aged 31–40, with secondary education)

Where doctors did not give information, some patients assumed that the doctors had a reason for not telling them anything:

‘I assumed that what they do not tell, I do not need to know’. (Female, aged 21–30, with primary education)

#### Theme 6: Language barrier

There were language barriers to effective communication. Patients reported that the healthcare practitioners spoke in English, sometimes using medical jargon:

‘I was unable to ask because the doctor was speaking in English – there was no one to translate, that's why I didn't seek clarity’. (Female, aged 41–50, with no formal education)

‘Medical terminology – most of the time doctors make use of big terms’. (Male, aged 21–30, with secondary education)

#### Theme 7: Fear of inconveniencing healthcare practitioners

Some patients indicated that they were fearful of the healthcare practitioners. Others worried about their questions being seen as ‘stupid’. This led them to refrain from seeking clarity where the healthcare practitioner's explanation was not understood:

‘I feel scared to ask doctors who appear to have a lot on their plates. I am even afraid to raise my concerns’. (Female, aged 51–60, with primary education)

#### Theme 8: The healthcare practitioner appeared uncertain about the patient's condition

Some patients indicated that they thought the healthcare practitioner was not sure of the patient's condition. These were the cases where the healthcare practitioner mentioned a few possibilities for the patient's condition and indicated that they were still investigating:

‘Doctors do not give me a direct answer, it's like they are also uncertain of my condition’. (Female, aged 21–30, with secondary education)

In some cases, patients found themselves confused by the various versions (sometimes conflicting) of their condition that were presented to them by different healthcare practitioners:

‘Different doctors are telling me different things, so I no longer ask where I do not understand, I'm just confused’. (Male, aged 21–30, with tertiary education)

### Patient-related themes

#### Theme 1: Notion that healthcare practitioners held patients in low esteem

The notion by patients that the healthcare practitioners held them in low esteem caused some patients to refrain from asking for clarity when it was necessary for them to do so. That notion was brought about by their perception that healthcare practitioners did not regard them as being worthy of consideration as they did not engage with the patients during their ward rounds. Furthermore, that notion led the patients to hesitate, as they were worried that their questions would be labelled as ‘stupid’:

‘How can you ask anything when they don't take you into consideration?’. (Male, aged 21–30, with secondary education)

‘[*Doctors*] they'll think you are stupid if you ask certain things’. (Male, aged 21–30, with secondary education)

#### Theme 2: Incapacitating clinical conditions

Some of the reasons given by patients for not asking questions about their clinical conditions related to the incapacity induced by the clinical conditions themselves:

‘I was too weak to talk’. (Male, aged 41–50, with tertiary education)

‘At first I was distraught that I had to deliver quickly so my stress was too much…’. (Female, aged 31–40, with secondary education)

#### Theme 3: No reason for not asking

It was noted that sometimes the inpatients could not give reasons for not asking for clarity – it just had not crossed their minds:

‘It didn't cross my mind to ask questions on their instructions to take the medicine’. (Female, aged > 60, with secondary education)

#### Theme 4: Patients’ fear of bad news

Some of the patients were reluctant to ask questions for fear of hearing bad news which would cause them distress:

‘I was afraid of seeking clarity on my diagnosis in fear that my condition may be serious’. (Female, aged 13–20, with secondary education)

#### Theme 5: Patients already knew their conditions

Some patients indicated that they had already known about their clinical conditions before admission (some had been informed by their family doctors):

*‘*I didn't seek clarity because I knew before I was admitted’. (Male, aged 31–40, with no formal education)

### Ethical considerations

Ethical approval was obtained from the MEDUNSA Research Ethics Committee (MREC) of the University of Limpopo (Clearance Certificate Number: MCREC/M/24/2008:IR). Permission to conduct the study on site was granted by the Chief Executive Officer (CEO) of the DGMAH. Ethical principles of confidentiality, justice and autonomy were ensured throughout the study. Personal details were not included on the questionnaires so as to ensure anonymity.

## Discussion

This study sought to explore the reasons given by inpatients for not seeking clarity on information received from healthcare practitioners on their clinical conditions. As far as the authors could ascertain, this was the first study in a healthcare institution in South Africa to examine this issue. The themes that emerged were related, on the one hand, to the inpatients’ perceptions of healthcare practitioners and, on the other hand, to the inpatients’ perceptions of themselves.

The perception that inpatients had that doctors and nurses were often rushed, contributed significantly to their not seeking clarity on the information they were given. As studies have shown, patients are reluctant to inconvenience the doctors that they perceive to be busy by delaying them with clarification questions.^12, 16^ However, this approach disempowers patients and renders them passive partners in the management of their own clinical conditions.^22^ The partnership with patients in the management of their conditions has been shown to be vital in patient care.^23^

Apart from doctors, nurses could also been seen as a source of information for patients. Nurses are perceived to spend more time with patients than doctors. That said, studies have shown that they spend less than 40% of their time ‘by the bedside’ of patients.^24, 25^ In our study, patients reported nurses as being ‘too busy’ to be consulted for clarification of information given to patients during the ward rounds. Nursing duties are multiple; besides taking ward rounds with doctors and other healthcare team members, they also have administrative tasks. These duties compete with direct patient care.^19^ Furthermore, in South Africa the patient to nursing ratio is unfavourable for prolonged interaction with patients in public healthcare institutions.^26^

The finding that inpatients were ‘told not to ask questions’ goes against the *Batho Pele* (‘Putting People First’) Principles^27^ advocated by the South African government toward improved accountability and efficiency in service delivery. The South African National Patients’ Rights Charter states that the patient has the right to be ‘attended to by only clearly identified health care providers’.^3^ Because of this right, any patient has the option to take the matter up with relevant hospital authorities on the identified healthcare practitioner. Withdrawing from interaction all together by ‘pretending to be fine’ should be discouraged,

Furthermore, inpatients perceived healthcare practitioners to be ‘distant’ from their patients. Healthcare practitioners need to be conscious of their body language when dealing with patients.^28^ The inpatients observed doctors discussing ‘amongst themselves’ and not with the patients. Thus, the message received by the inpatients was that the doctors were distancing themselves from the patients. The patients then adopted the attitude of ‘so it is up to them if they want to tell us anything’. That discouraged patients from asking anything of the doctors, even if they had overhead something they would have liked to have had explained. Such a tarnished relationship augurs badly for patient healthcare.^17^ Moreover, because the doctors were perceived to be unwilling to reach out to patients, they were regarded as ‘unapproachable’ – once again at patient's expense.

It is common practice in South Africa for healthcare practitioners who do not speak the patient's first language to make use of nurse interpreters.^29^ This has its own problems, in that the translation is not always accurate and more time is spent per patient using an interpreter compared with cases where there is no need for one.^30^ In this study, patients reported that healthcare practitioners spoke in English and sometimes used medical jargon during the clinical encounter. It has been recommended that healthcare practitioners have at least a minimum understanding of their patients’ languages and culture so as to facilitate effective communication.^31^ The use of medical jargon with patients constitutes ineffective communication. Resource material is available in order to assist healthcare practitioners with using ‘lay’ language with patients.^32–34^

Healthcare practitioners need to discourage the perpetuation of patients’ ideas about doctors being experts in their field who ‘know it all’, preventing patients from making enquiries about their clinical conditions. Patients need to be encouraged to ask questions since they are equal partners with healthcare practitioners in the management of their clinical conditions.^11^ Patients should be encouraged to use the encounter with their healthcare practitioners to empower themselves. With the advent of the internet, the paradigm is changing^35^ and healthcare practitioners need to prepare themselves to be engaged by patients regarding their clinical conditions.

Some patients expressed fear of inconveniencing their doctors – given their busy schedule – and were also reluctant to ask questions which they feared would be labelled as ‘stupid’. The power gradient between a patient and the healthcare practitioner, where the latter is the dominant figure, has been challenged in recent years.^36, 37^ However, a minority of patients have still been found to perceive doctors as authoritarian (16% in South Africa, 17% in the United States and 20% in the United Kingdom).^38^ The notion that the healthcare practitioner is an expert in the healthcare field has recently been counter-balanced by the realisation by healthcare practitioners that the patient is also an expert in the experience of his or her illness.^39^ This has paved the way for a negotiated solution between the two ‘experts’, thus diminishing the power gradient. Medical schools need to promote this paradigm shift in their training programmes, to ensure that the future of healthcare practice is not characterised by the ‘all-knowing healthcare practitioner’ who has little to learn from his or her patients, but a future where the healthcare practitioner and patients collaborate and negotiate for better health outcomes.

Some patients indicated that they did not ask for clarity when healthcare practitioners indicated that they were uncertain with regard to the patient's condition. However, studies have shown that patients develop confidence in healthcare practitioners who are honest enough to admit that they are uncertain about certain aspects of patient care, prompting them to search for the information for their patients.^40^ In our study, some patients had the notion that doctors ‘know what they are doing’, but others realised that the doctors ‘appeared not to know’ about their condition, whilst other inpatients became confused when there was ‘conflicting information’ from various doctors about their clinical conditions. Healthcare practitioners should avoid a situation where patients are confused by the conflicting information they receive from the healthcare practitioners in attendance. They need to share information amongst themselves about a given patient and endeavour to present a cohesive and comprehensive clinical picture to the patient.^41^ Furthermore, it is a challenge for healthcare practitioners, not only those at DGMAH, to educate patients about the fact that there is frequently a need for healthcare practitioners to search for information relating to their patients’ conditions and management, which is performed in the best interests of the patient.^42^

Regarding patient-related reasons for the inpatients not to seek clarity, it was of note that patients had a notion that healthcare practitioners held them in low esteem. That notion came as a result of their perception of how they were perceived by healthcare practitioners. They stated that healthcare practitioners ‘did not regard (them) worth considering’. This could have been a combination of other factors already mentioned above, *inter alia*, the aloof stance toward patients that healthcare practitioners were seen to adopt, healthcare practitioners being too busy for patients and the negative attitude that they were perceived as having toward patients. Patients’ perceptions of a negative attitude on the part of healthcare practitioners, especially for chronic diseases, has been documented.^43^

Those inpatients who had no reason for not asking for clarity on areas they did not understand presented a challenge to the healthcare practitioner to always invite questions following each healthcare practitioner–patient encounter.^12^ Some inpatients indicated that they were not keen to seek clarity on their condition because they feared hearing bad news from the healthcare practitioners. Patients have been found to suffer from reactive depression following their hearing bad news, necessitating referral for psychosocial counselling.^44^ However, withholding information from patients on the ground of their fear of bad news is not advised. Healthcare practitioners should therefore address their patients’ fears and concerns during the breaking of bad news so as to help patients overcome this particular barrier.

Some patients indicated that they had already known about their clinical conditions before admission, hence they had seen no need to seek further clarity. It is highly unlikely that information received by a patient about their condition prior to admission was exhaustive. To this end, at the end of every communication with a patient, the healthcare practitioner should invite questions from the patient and make sure the patient indeed understands.^12^

### Limitations of the study

The study did not specify the various cadres of the healthcare team (nurses, doctors, healthcare science personnel, etc.) to whom patients were referring in their responses. This lack of specificity makes it difficult to direct specific feedback to the relevant healthcare teams. There were no follow-up questions to establish a deeper understanding of the inpatients’ responses. Errors could have been introduced during the translation of patients’ responses from their mother tongue to English. This was minimised by the involvement of the expert in Linguistics in the translation.

The study was conducted in a tertiary institution, which limits its transferability to other settings. Nevertheless, tertiary hospitals as ultimate referral institutions should demonstrate a high standard of healthcare, including healthcare professional–patient relationships. Furthermore, revealing the socio-cultural and economical demographics of the participants could have helped the reader to judge the applicability of this research in other settings and could have helped with regard to the outlining of possible correlations and trends.

### Practical implications and recommendations

Asking for clarification of information received by inpatients from healthcare practitioners is pivotal for an effective healthcare practitioner–patient encounter and will lead to greater understanding and therefore adherence to mutually-agreed therapies. The study has described, from the inpatient's perspectives, some of the factors that stand in the way of an effective two-directional communication between healthcare provider and patient. Healthcare professionals should make a conscious effort to develop attitudes and skills that motivate patient engagement through effective communication in plain language, which should include inviting the patients to ask questions after each healthcare professional–patient encounter as well as asking them to express their specific fears and concerns.

## Conclusions

Reasons given by patients for not seeking clarity about their clinical conditions at DGMAH were based mainly on patients’ perceptions of healthcare practitioners, their clinical conditions and perceptions of themselves. They perceived healthcare practitioners to be ‘too busy’, aloof, non-communicators and sometimes uncertain about patients’ conditions. Although some had unquestioning trust in their healthcare practitioners others had experienced bad treatment from them. They had incapacitating clinical conditions, fear of bad news and an impression of adequate prior knowledge of their conditions. Some had the notion that healthcare practitioners held them in low esteem.

Future studies should investigate challenges faced by healthcare practitioners in a healthcare practitioner–patient encounter which are barriers in this encounter. Other future research could focus on factors that motivate patients to ask for clarification where they did not understand.
